# Behavior of QQ-Plots and Genomic Control in Studies of Gene-Environment Interaction

**DOI:** 10.1371/journal.pone.0019416

**Published:** 2011-05-12

**Authors:** Arend Voorman, Thomas Lumley, Barbara McKnight, Kenneth Rice

**Affiliations:** Department of Biostatistics, University of Washington, Seattle, Washington, United States of America; University of Hong Kong, Hong Kong

## Abstract

Genome-wide association studies of gene-environment interaction (GxE GWAS) are becoming popular. As with main effects GWAS, quantile-quantile plots (QQ-plots) and Genomic Control are being used to assess and correct for population substructure. However, in G
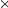
E work these approaches can be seriously misleading, as we illustrate; QQ-plots may give strong indications of substructure when absolutely none is present. Using simulation and theory, we show how and why spurious QQ-plot inflation occurs in G
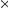
E GWAS, and how this differs from main-effects analyses. We also explain how simple adjustments to standard regression-based methods used in G
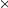
E GWAS can alleviate this problem.

## Introduction

Genome-wide association studies of Gene-environment interaction (G
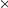
E GWAS) are now being undertaken to search for modification of environmental effects by genotypes [1], [2]. As in main-effects GWAS that search for the effects of genotype alone, differences in recent ancestry, termed population substructure, can be mistaken for true genetic effects, and is therefore a serious concern [1], [3].

In main-effects GWAS, the extent of the substructure problem is typically addressed using Genomic Control [4]. Here, under the assumption that processes of local mating and genetic drift inflate measures of association in the same way genome-wide, the degree of inflation of the median test statistic (known as 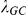) is a useful assessment of the degree of test statistic inflation at all levels. Dividing test statistics by 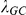 is a widely-used approach to correct for minor substructure problems; for examples, see e.g. [5], [6]. Adjusting for principal components, which we will use in this paper, is another popular correction method [7], [8].

In G
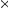
E GWAS, one can also argue that substructure leads to inflation of test statistics by a multiplicative factor. However, in G
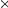
E GWAS the same inflation can also be caused by an entirely different mechanism: systematic underestimation of variability of effect estimates across the genome. This is not confounding, but it gives the appearance of confounding; hence nave use of Genomic Control can be misleading.

In this paper, we show how the separate effects of population substructure and underestimation of variability affect interpretation of G
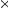
E GWAS results, and we show how this problem can be solved. In the Results section, using simulation and theory, we describe how spurious QQ-plot inflation can occur. We also illustrate how model-robust estimates of standard errors (also known as “sandwich” standard errors) rectify the problem, while retaining 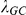
's ability to identify true substructure.

### Assumptions in G
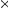
E GWAS: classical approaches

In general, regression methods incorporate assessments of variability by estimating standard errors; for a given estimated effect (i.e. 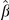), larger standard errors reflect greater variability from sample to sample, and produce less significant results. However, the precise assumptions reflected in these statements of variability differ between methods.

Under “classical” or “model-based” regression approaches, standard errors only account for random variation in the phenotype (denoted 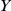). Furthermore, for their validity these classical variability estimates require that the mean value of 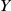 is truly linear in the coefficients of the independent variables, such as environmental variables (denoted 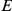) or genotypes (denoted 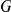) [9].

To illustrate these classical assumptions, we consider linear regression, with 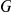 coded as 0/1/2 copies of the minor allele. For classical main-effects analysis one might assume that the mean value of 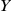 truly is
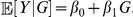
Association would be assessed using the least squares regression estimator 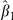 and its estimated standard error, which is based on estimated random variation in the phenotype 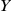 with the values of the observed predictor 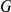 fixed. (Formally, the analysis is ‘conditioned’ on the independent variable 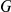) [10].

Using the classical approach for interaction analyses, one might instead assume that
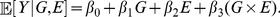
(1)Inference would use 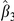 and its estimated standard error, where again the variability accounted for by model-based standard errors is that of the phenotype, 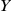, in replicate experiments where 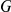 and 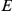 are fixed at the values observed in the original data.

How does the mean model assumption affect GWAS work? In main effects analyses, the validity of the mean model is not a major concern. Under the ‘strong null hypothesis' of no association between 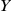 and 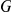, the true mean value of 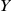 is simply
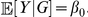
This means that the model assumptions hold under the null hypothesis, which is sufficient for valid p-values. But in G
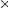
E work, even under the null hypothesis of no statistical interaction (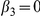 in (1)), model-based standard errors assume that the mean of 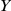 is truly linear in 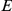 and the residual variance is constant with respect to 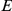. When this assumption fails, model-based errors may be too small.

How does accounting for different sources of variability impact GWAS work? In main-effects analyses, we typically have the same, well-specified model for each gene we test, under the null hypothesis. In this case, the variability in our estimates is the same whether or not 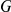 is truly fixed. As a result, model-based standard errors can be used to produce valid QQ-plots, even though each point on the plot represents a different 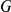. But when there is mean-model mis-specification in G
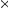
E GWAS, variability in interaction term coefficient estimates from 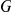 to 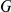 becomes important. QQ-plots using model-based standard errors provide results based on viewing 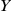 as random, and 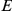 and 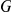 as fixed. This contrasts with the observed variation in p-values entering the computation of 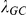, where 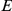 is fixed, but 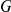 varies – all along the genome. In particular, this means that 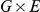 varies in a way not accounted for by model-based analysis.

We will see that in G
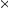
E GWAS using model-based standard errors, the behavior of QQ-plots and 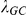 may not be as straightforward as in main-effects work. In Results, we show how violation of the assumptions both about mean-model validity and what is considered random can lead to misbehaved QQ-plots in 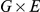 studies.

### Assumptions in G
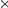
E GWAS: robust approaches

‘Model-robust’ standard errors are an alternative to model-based. Here, instead of assuming a particular form for the mean 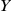 given 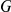 and 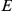, standard error estimation views regression estimates as simple summaries of the observed association between 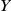 and 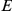, or 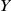 and 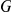. For example, interaction terms summarize how a measure of the 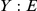 association differs between values of 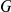. While the summary is expressed linearly, no underlying assumption of true linearity, in either the 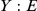 relationship or how it differs between levels of 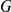, is required for accurate standard error estimates [11]. Thus, concerns about mis-specification of the mean model in G
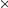
E GWAS disappear. This form of standard error estimation should give inherently better-behaved QQ-plots than the model-based approach.

Model-robust standard error estimates are known as “heteroscedasticity-consistent”, “model-agnostic”, “Huber-White”, or “sandwich” standard errors, and are available in standard statistical software [12]–[14]. Unlike model-based standard errors, they summarize uncertainty in estimates where 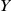 and all independent variables are considered random. In 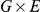 GWAS work, this means that repeated sampling variability in 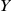, 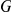 and 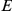 is accounted for. However, when we examine QQ plots we have 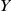 and 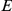 fixed while only 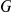 varies. As will be discussed in the theoretical portion of the Results, this produces about the same amount of variability as when all variables are considered random, and more than when only 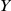 is considered random. As a result, robust standard errors should give a better assessment of variability than model-based standard errors when we vary 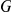 due to genome-wide comparison as we do on a QQ-plot.

## Results

### Simulation results

Before deriving theoretical results, we illustrate the scope of the difference between model-based and model-robust inference in G
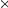
E GWAS, and the extent of QQ-plot inflation that may be produced in the absence of population substructure.

**Figure 1:**
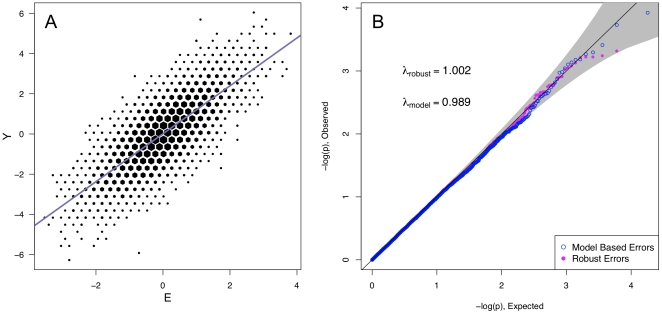
Correctly Specified Model. In this scenario the data is generated according to 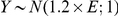, independent of 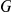. Both the model-based and robust standard errors are valid estimates of variability, as demonstrated by the QQ-plot.

**Figure 2:**
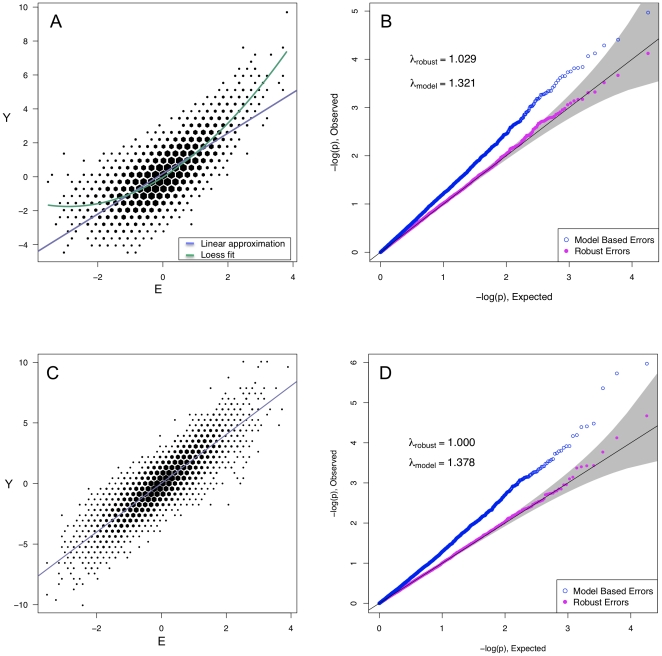
Mis-specified model. Panels A and C show scatterplots of 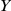 vs. 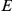 generated according to 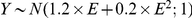 and 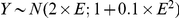 respectively, independent of 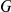. Panels B and D demonstrate the corresponding effect of this mis-specified mean model and non-constant variance.

In Figures 1 and 2, we show the QQ plots for linear regression results in G
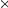
E GWAS, based on simulations of well specified and misspecified modeled relationships between 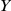 and 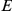. All simulations use Wald tests, independent Normal phenotypes 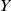, biallelic genotypes 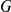 in Hardy Weinberg equilibrium with MAF varying between 0.02 and 0.5 and coded as 0/1/2 copies of the minor allele; for details see Methods. Importantly, the null hypothesis of no 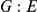 interaction holds throughout, and no population substructure is present. Using model-based standard errors, in Figure 1 we see no inflation beyond that expected by chance alone. In Figure 2, in the presence of either of two types of slight model mis-specification, substantial inflation of model-based statistics is observed (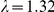 and 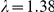), well beyond chance, despite the absence of real interactions or of population substructure. Using the model-robust approach, we see no inflation in the correctly specified model (Figure 1), or for either of the mis-specified models (Figure 2).

**Figure 3:**
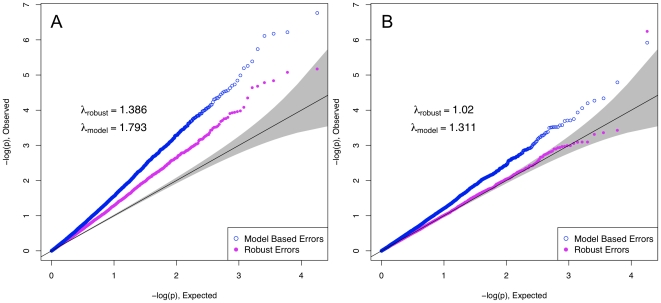
QQ-plots with added population structure. In the left panel, nothing is done to account for the structure. On the right, the results are adjusted for principal components, leaving about the same amount of inflation as the case with no population stratification.

In Figure 3, we show that similar behavior can occur when substructure is present in an interaction analysis with model mis-specification. Here, structure was incorporated by assigning MAFs to two sub-populations, choosing Wright's 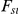 to be 0.01, and the mis-specification exactly that displayed in panels A and B of Figure 2. Using a model-based analysis that accounts for the substructure by including one principal component of the SNP data as a covariate in the regression, we see that inflation persists, spuriously. However, the principal component-adjusted model-robust inference removes the substructure problem, and again gives correctly-calibrated 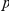-values.

**Figure 4:**
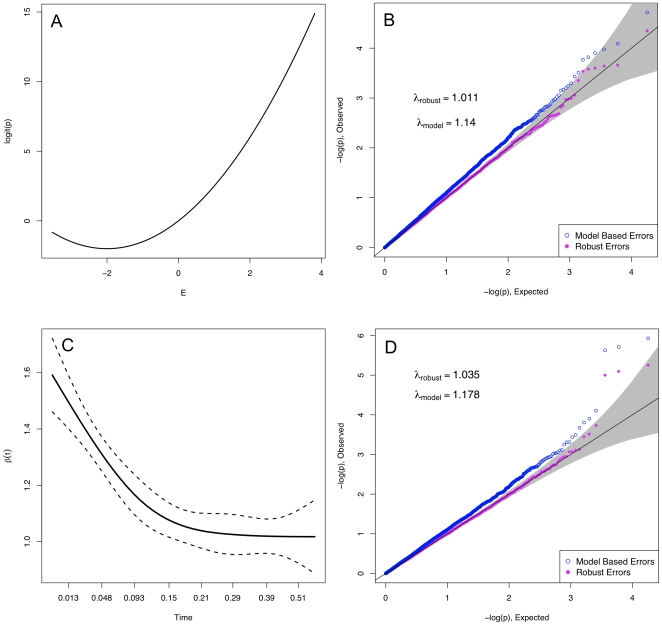
Example of behavior in logistic and proportional hazards regression. The top row displays the results for logistic regression, and the bottom for proportional hazards. The data was simulated according to 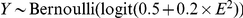 and 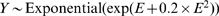 with half of the data censored at the median survival time. The top left shows the log odds of an event, which demonstrates non-linearity that was not specified in the model. The plot on the lower left displays a loess curve through the Schoenfeld residuals from the regression of Y on E. A non-zero slope is indicative of violation of the proportional hazards assumption.

Finally, in Figure 4, we show that similar behavior holds for non-linear regression analysis. In these, model-based errors assume linearity on a modified scale: logit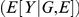 for logistic regression, and the log hazard for Cox proportional hazards regression. Here, in the top row we show results for binary 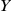, a 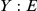 relationship that is non-linear on a logit scale, and no true interaction. In the bottom row, we show similar results for a mis-specified Cox proportional hazards regression, with uniform censoring at the median [15]. Similar results hold when using likelihood ratio tests and joint tests of 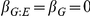.

### Theoretical results

We now develop theoretical results governing the behavior of 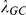 under model-based and model-robust analyses of G
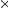
E GWAS.

In the absence of population structure, the population parameter consistently estimated by 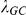 for interaction terms can be viewed as a ratio of conditional and unconditional variances, as follows:
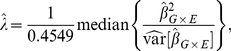
(2)where .4549 is the median of the 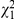 distribution and 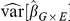 is the variance estimate, either model-based or robust, used in the analysis. For simplicity we first consider the situation where 1) 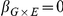 for all 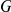, where 2) 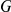 is independent of 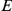, and where 3) the minor allele frequency is the same for all SNPs 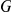. We note that, in the absence of population stratification, the first two conditions are approximately true for nearly all SNPs. The third condition will later be relaxed. Under these three conditions, 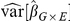 is approximately constant and can be factored out of the computation of the median in equation (2).

Since 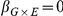 and 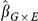 is asymptotically Normal,
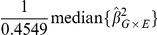
is consistent for the variance of 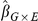 taken over the distribution of 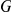 but conditioning on 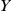 and 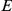. The genomic control 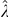 can then be written as
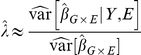



The numerator of 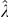 is the empirical variance of the regression coefficients and is always a good estimate of 
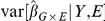
, the true variance over genotypes fixing the outcome and exposure variable. The denominator of 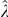 is the estimated variance of 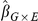 from the regression analysis. If model-based inference is used, this estimates 
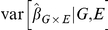
, the variance taken over the distribution of the outcome, conditional on the predictor variables. If a model-robust variance estimator is used, the denominator estimates 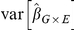, the unconditional variance of 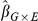 taken over the distribution of all variables.

To see that 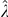 should be approximately 1 when there is no population structure, despite the conditioning on 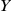 and 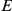 that is implicit in the computation of its numerator, we can examine the variance decomposition:
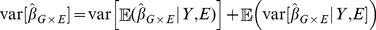
(3)The numerator of 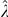 accurately estimates the second term in this decomposition. We show in Appendix S1 that the first term is approximately zero for the case of linear regression, so
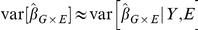
as required. Our simulations confirm that this result also holds for logistic regression and Cox regression.

So far we have assumed constant MAF, but the arguments do not depend on the value of the MAF, nor does the conclusion that 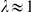. Since 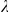 is defined from the median of the chi-squared statistic, if 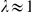 for the SNPs with each fixed MAF we must also have 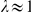 pooling over a range of MAF. For this reason, the results should hold with typical range of MAFs seen in GWAS so long as the sample size and MAF are large enough to allow accurate estimation of the sandwich variances. This is further supported by the simulation results, which used a wide range of MAFs.

**Figure 5:**
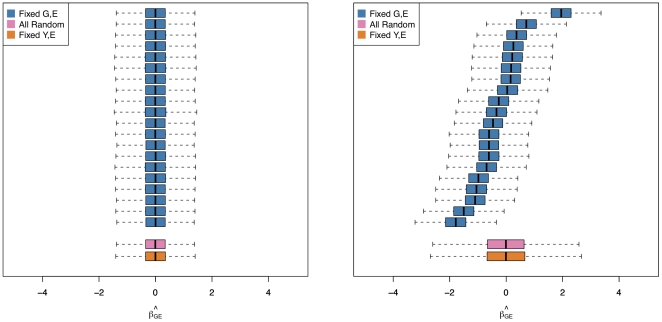
Illustrating the variance decomposition. The panels show estimates of 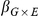 over replications with different variables held constant. At left, the 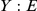 relationship is truly linear. Because 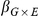 is the same regardless of which variables are held constant, then according to the variance decomposition, so is the variability. In the right panel the 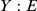 relationship is exponential. With 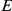 and 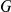 fixed, a certain amount of within-sample correlation remains fixed, making 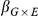 different for each instance of 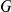. Both the 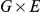 setting where 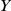 and 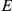 are fixed and 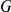 is random, and the setting when all variables are random incorporate this extra variability.

The analog of equation 3 for the model-based estimator is
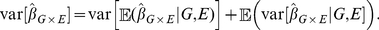
(4)The first term in this decomposition is not negligible unless the 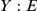 model is correctly specified, so under model mis-specification
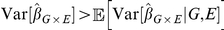
and 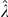 will tend to be greater than 1 even when there is no confounding by population substructure. Figure 5 shows an example of this.

As a further complication, the model-based variance estimator 
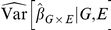
 need not be close to the true variance, the second term in equation 4, if the model is misspecified [16].

## Discussion

We have seen in the above that standard errors that rely on model assumptions can be underestimates of 
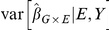
 when those model assumptions are not met, while model-robust estimates of variance provide well-calibrated standard errors and p-values. This distinction can be seen in all types of regression examined. The problem is not merely theoretical; our research was motivated by seeing apparent population substructure similar to that in Figure 2 in initial analyses of a 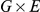 GWAS of echocardiographic traits [17] and noticing that the inflation was absent in cohorts that had used model-robust standard error estimates. The simulation results from linear regression show that even mild heteroskedasticity or mean-model mis-specification can inflate model-based test statistics.

### Intuitively explaining sources of variability

The impact of different sources of variability and its relation to model mis-specification is not well recognized. We illustrate the situation for 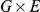 GWAS in 5. Here, for a continuous phenotype 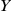, continuous exposure 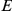, and binary genotype 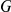, we show the spread of 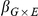 estimates holding different variables constant when there is no true interaction or population structure present. Within the blue boxes, 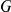 and 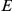 are held fixed while 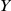 is varied to produce different estimates of 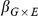. From boxplot to boxplot 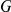 is varied. Each blue boxplot illustrates the variability in 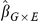 using what model-based errors assume is fixed; it can be compared to the variability with 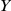, 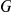, and 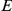 all random, and with 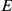 and 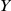 fixed. Under model mis-specification, it is clear that the distribution of 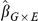 varies from 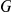 to 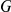, and that the variability in 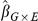 is larger when 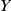, 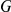 and 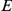 are all random or when only 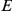 and 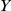 are fixed.

When the linear model is true, as in the data summarized in left panel, then the linear trend is the same for any level of 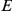. When this is true, the variability in 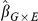 is the same whether or not 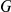 and 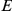 are taken to be random. However, when the linear model is not true, then the linear trend need not be the same at different levels of 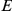. In right panel of Figure 5, the data were generated according to an exponential relationship between 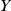 and 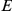. Under this model the linear trend will be steeper in samples where the values of 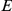 are larger. Now for any single instance of 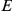 and 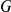 there is always some small degree of correlation between them within the data. As each of these small, fixed associations between 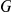 and 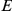 varies over 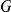, there is truly effect modification: subjects with different genotypes will tend to have slightly different levels of 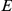, and hence a slightly different relationship with 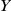. So in addition to the usual sampling variability in estimating 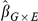, we have this ‘bias’ that varies from each pair of 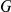 and 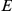 to the next. If we add these two sources of variability, we obtain the full variability that we observe when 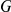 and 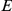 are also random.

### Conclusions

In G
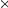
E GWAS, nave use of QQ-plots and genomic control with model-based standard errors may lead to false conclusions about substructure. The extent of this problem depends on the degree of mis-specification of the mean-model, the form of regression used, and the distribution of the environmental exposure. Use of model-robust inference offers a simple alternative that avoids these difficulties, and retains genomic control as a useful tool for the assessment of substructure.

## Methods

Simulation studies in R [18] were used to assess the performance of model-based standard errors and sandwich standard errors in a variety of scenarios, with the genomic-control 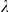 used to assess the degree of inflation in the test statistics. Visually, this can be seen in QQ-plots.

We simulated a normally distributed environmental exposure, and a response generated from this either under a correctly specified linear model, or under a quadratic mean-model. Genotypes at 10,000 loci were simulated according to a binomial distribution, with minor allele frequency (MAF), drawn from a beta(.5,.5) distribution truncated at 1/2, and with frequencies filtered to be above 0.02. We found that the behavior of the simulations was not affected in a substantial way when the MAF was fixed at any particular value for all loci. In this way, genotype is entirely unrelated to phenotype in these simulations, and so we would hope that tests for gene-environment interaction yield uniformly distributed p-values, as they should be under the null hypothesis.

Population stratification was simulated by drawing an MAF for each of two sub-populations at each locus, centered around some MAF drawn from the distribution described above. These sub-population MAFs were distributed according to a beta distribution parametrized by the central MAF and Wright's 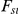, in this case chosen to be 0.01 [4]. In order to allow for confounding, we created a slight difference in the relationship between phenotype and environmental exposure: the linear component of the relationship was 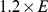 20% of the population and 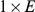 in the other population, while the quadratic component was 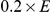 in both groups.

In addition to linear regression, performance of model-based and sandwich standard errors was assessed in logistic and proportional hazards regression. In these situations we generated simulations in which departures from linearity were on the appropriate transformed scale. In logistic regression, this meant that the linearity was judged on the scale of the logit of the probability of ‘success’. In proportional hazards, the scale was on the log hazard scale. To achieve this, we generated exponentially distributed event times where the exponentiated ‘rate’ parameter was related quadratically to exposure.

## Supporting Information

Appendix S1 (PDF)
