# Supplementary material for: Behavior of QQ-Plots and Genomic Control in Studies of Gene-Environment Interaction
Source: PLoS One. 2011 May 12;6(5):e19416. doi: 10.1371/journal.pone.0019416 (PMC3093379; doi:10.1371/journal.pone.0019416)
Supplement: Appendix S1 — (PDF) [file pone.0019416.s001.pdf]

Appendix S1

For the case of simple linear regression, we can show directly that $\mathbb{E}[\hat{\beta}_{G\times E}|Y,E] \approx 0$ with high probability (ie, for virtually all realizations of Y and E). Suppose we work with centered variables: $E-\bar{E}$, $G-\bar{G}$, and $(G-\bar{G})(E-\bar{E})$. Let X be the design matrix with columns $(1,\ E-\bar{E},\ G-\bar{G}$, and $(G-\bar{G})(E-\bar{E}))$ and write

$$\hat{\beta} = (X^TX)^{-1}(X^TY).$$

Consider the interaction component of X^TY, which is $\sum_{i=1}^n (G_i-\bar{G})(E_i-\bar{E})Y_i$. This is linear in G, so its conditional expectation given E and Y is just

$$\sum_{i=1}^N \mathbb{E}[G_i-\bar{G}|E,Y](E_i-\bar{E})Y_i = \sum_{i=1}^N 0\times(E_i-\bar{E})Y_i = 0.$$

The same is true of the genetic main-effect component, which is $\sum_{i=1}^n (G_i-\bar{G})Y_i$ and has expectation

$$\sum_{i=1}^N \mathbb{E}[G_i-\bar{G}|E,Y]Y_i = \sum_{i=1}^N 0\times Y_i = 0.$$

By a similar argument, $\mathbb{E}[X^TX|Y,E]$ has the diagonal form

$$\mathbb{E}[X^tX] = \begin{bmatrix} n & 0 & 0 & 0 \\ 0 & \sum(E_i-\bar{E})^2 & 0 & 0 \\ 0 & 0 & (n-1)\mathrm{var}[G] & 0 \\ 0 & 0 & 0 & \frac{n-1}{n}\mathrm{var}[G]\sum(E_i-\bar{E})^2 \end{bmatrix}$$

where the first row and column are zero due to centering and the remaining off-diagonal terms are zero due to independence of G and E. Thus $\mathbb{E}[X^TX]^{-1}$ is also diagonal. This means that the G and $G\times E$ entries of

$\mathbb{E}[X^TX|Y,E]^{-1}\mathbb{E}[X^TY|Y,E]$ are zero. Omitting SNPs where X^TX is singular (eg, those that are monomorphic in the sample),

$$\begin{aligned}\hat{\beta} &= \mathbb{E}[(X^TX)^{-1}X^TY|Y,E] \\ &= \mathbb{E}[X^TX|Y,E]^{-1}\mathbb{E}[X^TY|Y,E] + O_p(1/n) \\ &= O_p(1/n)\end{aligned}$$

so $\mathbb{E}[\,\hat{\beta}_{G\times E}|Y,E\,] = O_p(1/n)$ and $\mathrm{Var}\left[\mathbb{E}[\,\hat{\beta}_{G\times E}|Y,E\,]\right] = O(1/n^2)$.

The second term in equation (3) is $O(1/n)$, so the first term is of smaller order and can be ignored when sample size is large. A model-robust variance estimator will give $\hat{\lambda} \approx 1$ in the absence of population substructure.
